# Prenatal detection of a 7q11.21 microdeletion (517–605 kb): A variant with normal characteristics at birth (STROBE)

**DOI:** 10.1097/MD.0000000000024560

**Published:** 2021-02-12

**Authors:** Hongguo Zhang, Leilei Li, Yang Yu, Linlin Li, Yuting Jiang, Ruizhi Liu

**Affiliations:** a Center for Reproductive Medicine, Center for Prenatal Diagnosis, First Hospital, Jilin University; b Jilin Engineering Research Center for Reproductive Medicine and Genetics, Jilin University, Changchun, China.

**Keywords:** 7q11.21 microdeletion, prenatal diagnosis, single nucleotide polymorphism array

## Abstract

In the literature, 7q11 deletion was reported with various abnormalities. However, there were other genetic conditions combined with 7q11.21. It is necessary to have sufficient pure 7q11.21 microdeletions for classifying the pathogenic categories of variation.

Chromosomal karyotyping analysis was performed on cultured amniotic fluid cells. Eighteen pregnant women took chromosomal microarray using prenatal amniotic fluid samples at our center by Affymetrix CytoScan750K_Array. We followed the outcome of these pregnancies and determined postnatal health conditions.

Cytogenetic studies delineated that all patients had normal karyotypes. The exception was P17, who had 47, XN. Single nucleotide polymorphism array results showed 517 to 605 kb deletions of 7q11.21 (chr7: 64543313-65196780) in these cases. The microarray results were pure or combined 7q11.21 microdeletions. In 11 pure 7q11.21 microdeletions and 7 combined cases, there was no apparent abnormal phenotype associated with partial 7q11.21. Among them, only mothers of P10 and P17 decided to terminate the pregnancies due to 18 trisomy or ultrasound abnormal fetal strephenopodia. In the follow-up survey, the newborns had no apparent abnormalities.

In this study, we described 11 pure and 7 combined 7q11.21 microdeletions associating with no apparent postnatal phenotypic abnormalities. From this study, we can learn that the partial 7q11.21 deletion (chr7: 64543313-65196780) might be benign and have no association with human disorders.

## 1 Introduction

Low copy repeats, which are also called segmental duplications, are highly-homologous sequences (larger than 95% identity) comprising about 4% to 5% of the human genome.^[1]^ Due to the sequences similarity, the nonallelic homologous recombination happening during the meiosis results in copy-number variations (CNVs). In the haploid human genome, around 2% sequences show 100% identity.^[2]^ Since these regions shared sequences with about 80% known CNVs, it proves the contribution of DNA identity to nonallelic homologous recombination and CNVs formation.^[3]^ 7q11.21 microdeletion is a recurrent CNV formed by this mechanism.^[4]^

The interstitial 7q deletions include 7q11.22 associated with intellectual disability/developmental delay and 7q11.23, which cause Williams–Beuren syndrome (OMIM 194050)^[5,6]^ in Online Mendelian Inheritance in Man. There are case reports about patients with a deletion of 7q11.21-q11.23 and infantile spasms.^[7,8]^ However, the patient with pure 7q11.21 microdeletion was rare. The 461 kb 7q11.21 deletion was reported as a benign copy in 1 patient because it has a similar frequency in the control population.^[9]^ In chromosomal deletion map of human malformation, 7q11 were involved as part of “malformation-associated bands”, like 7q11-21 associated with choanal atresia and inguinal hernia, and 7q11-22 related to split hand.^[10]^ Based on the current reports, the genotype-phenotype correlation of pure 7q11.21 microdeletion is not easy to conclude. More cases with pure 7q11.21 microdeletion without other genetic conditions are essential for further research and clinical study.

Although conventional karyotyping is the gold standard in prenatal diagnosis, chromosomal microarray (CMA) is a powerful diagnostic tool. The advantages of CMA include high sensitivity, using uncultured material, and time-saving. Submicroscopic CNVs cause 5.2% to 10% ultrasound anomalies in fetuses with a normal karyotyping.^[11–13]^ Compared with karyotyping, CMA analysis can detect additional submicroscopic CNVs. In this way, CMA assists clinicians in finding additional clinical value in fetuses with a normal karyotyping. Single nucleotide polymorphism (SNP) array in this study is one CMA platform that can identify allelic imbalances, triploidy, low-level mosaics, and homozygosity.^[14]^

Here, we describe 18 pregnant women with interstitial 7q11.21 microdeletion with various indications for prenatal diagnosis and review related clinical data focusing on similar genetic conditions.

## 2 Methods

### 2.1 Patients

This study included 18 pregnant women with different clinical indications for amniocentesis recruited from May 11th, 2017 to September 17th, 2019. Their prenatal diagnosis showed pure or combined interstitial 7q11.21 microdeletions at the Center for Prenatal Diagnosis of First Hospital of Jilin University. We followed up the children of these pregnancies and determined their postnatal health conditions. The first follow-up survey was on December 2nd, 2017, while the last follow-up survey was on December 22th, 2019 (mother was still in pregnancy). All couples are not consanguineous marriage and without a family history of genetic disease. The pregnant women were healthy and did not take any medicine. Besides, they were no smoking, no drinking, no exposure to other toxic substances, or radiation. The Ethics Committee of the First Hospital of Jilin University approved this study. The patients for the study signed appropriate voluntary written consent and the experiments in this paper followed the standard operation procedure of the hospital.

### 2.2 Cytogenetic analysis

Routine chromosome analysis was performed on G-banding techniques at 300 to 400 banding resolution prepared from the cultured amniotic fluid cells according to standard protocols. Twenty metaphases were analyzed for all samples. The International System for Human Cytogenetic Nomenclature 2013 was used to describe the karyotype.^[15]^ Then couples were taken samples for karyotyping because of the abnormal SNP array results of the fetus after signing written informed consent. All couples, all fathers and mothers, reported to the doctors during genetic counseling that they are mentally and physically well (data not shown).

### 2.3 The SNP array

Genomic DNA was extracted using DNeasy Blood & Tissue Kit (Qiagen, GmBH, Germany), referring to the manufacturer's instructions. Then, the DNA was quantified using NanoDrop ND-2000 spectrophotometer (Thermo Fisher, Waltham, MA). The CNVs were detected by Affymetrix CytoScan750K_Array (Affymetrix, Santa Clara, CA). DNA digestion, ligation, fragmentation, labeling, hybridization, staining, and scanning were performed following the Affymetrix protocol. The data were analyzed with the software Chromosome Analysis Suite (Affymetrix). The reference database, including Database of Genomic Variants (GRCh37/hg19), OMIM, and DECIPHER, evaluated the array data and linked genotype-phenotype correlations.

## 3 Results

In our report, 18 patients with partial 7q11.21 deletion, including 11 pure interstitial 7q11.21 microdeletions, were identified by SNP array. The G-banding analysis showed that all fetuses presented normal karyotype results on 7q. However, SNP array successfully identified microdeletions on 7q11.21 in all cases. All cases shared a similar deletion of the ZNF92 locus (chr7: 64838712-64866038). The distributions of indications for prenatal diagnosis were as follows: advanced maternal age (7/18), high-risk noninvasive prenatal testing results (3/18), Down syndrome risk in maternal serum screening (2/18), increased nuchal translucency (2/18), other ultrasound abnormalities (5/18), and abnormal childbearing history (1/18). The cytogenetic, SNP array results, and clinical manifestations are listed at the end of Table 1 for all cases. Among the fetuses in this study, the smallest 7q11.21 microdeletion was 517 kb, while the largest was 605 kb.

**Table 1:** Summary of the cytogenetic, SNP array, and clinical findings of our cases with 7q11.21 deletion.

				Birth							Prenatal diagnosis indications/reason of study	Follow-up outcome
Case #	Sex	Pregnancy history	Gestation age	Weight (kg)	Length (cm)	Karyotype results	SNP array results	7q11.21 deleted size(kb)	De novo/inherited	Deleted genes on 7q11.21		
P1	F					46, XX	arr[GRCh37]7q11.21 (64679561-65196780)×1 (likely benign)	517	N.A.	INTS4L1,INTS4L2,RSL24D1P3,ZNF92	Fetal abdominal cystic echo, right renal parenchymal echo enhancement, circular of umbilical cord	No apparent abnormalities
P2	M	G4P1A1	40W + 2	4.25	53	46, XY	arr[GRCh37]7q11.21 (64612879-65148399)∗1 (VOUS)	536	N.A.	INTS4L1,INTS4L2,RSL24D1P3,ZNF92	DS:1/246	No apparent abnormalities
P3	M	G4P1	38W + 1	2.9	50	46, XY	arr[GRCh37]7q11.21 (64612879-65148399)∗1 (VOUS)	536	N.A.	INTS4L1,INTS4L2,RSL24D1P3,ZNF92	Advanced maternal age	No apparent abnormalities
P4	F	G2P1	38W	3.45	52	46, XX	arr[GRCh37]7q11.21 (64612879-65148399)∗1 (VOUS)	536	N.A.	INTS4L1,INTS4L2,RSL24D1P3,ZNF92	high risk NIPT result of chromosome 5	No apparent abnormalities
												
P5	F	G1P0	39W + 6	3.16	50	46, XX	arr[GRCh37]7q11.21 (64543313-65148399)∗1 (VOUS)	605	N.A.	INTS4L1,INTS4L2,RSL24D1P3,ZNF92	DS:1/540, NIPT 13 trisomy high risk	No apparent abnormalities
P6	M	G1P0	35W	2.4	43	46, XY	arr[GRCh37]7q11.21 (64568823-65162169)∗1 (VOUS)	593	N.A.	INTS4L1,INTS4L2,RSL24D1P3,ZNF92	Advanced maternal age	No apparent abnormalities
												
P7	M	G4P1	38W + 2	3.5	52	46, XY	arr[GRCh37]7q11.21 (64543313-65148399)∗1 (VOUS)	605	N.A.	INTS4L1,INTS4L2,RSL24D1P3,ZNF92	Increased NT	No apparent abnormalities
												
P8	F	G2P0	41W + 1	3.65	51	46, XX	arr[GRCh37]7q11.21 (64612879-65148399)∗1 (VOUS)	536	N.A.	INTS4L1,INTS4L2,RSL24D1P3,ZNF92	Right kidney dysplasia in fetal ultrasound	No apparent abnormalities
												
P9	M	G2P1	38W	3.25	50	46, XY	arr[GRCh37]7q11.21 (64576417-65148399)∗1 (VOUS)	572	N.A.	INTS4L1,INTS4L2,RSL24D1P3,ZNF92	Advanced maternal age	No apparent abnormalities
P10	N.A.	G3P1A1	N.A.	N.A.	N.A.	46, XY	arr[GRCh37]7q11.21 (64576417-65148399)∗1 (VOUS)	572	N.A.	INTS4L1,INTS4L2,RSL24D1P3,ZNF92	Fetal strephenopodia in ultrasound	terminated pregnancy
P11	M					46, XY	arr[GRCh37]7q11.21 (64679561-65196780) × 1 (likely benign); 11p11.12 (51193615-51530241) × 3 (likely benign); 11q12 (54835623-55379944) × 3 (likely benign)	517	Paternal inherited	INTS4L1,INTS4L2,RSL24D1P3,ZNF92	Variation of aortic arch branches in fetal ultrasound	No apparent abnormalities
P12	M	G1P0	39W + 2	3.85	49	46, XY	arr[GRCh37]5q35.3 (178746631-178936592)∗3 (VOUS)/arr[GRCh37]7q11.21 (64568823-65148399)∗1 (likely benign)/arr[GRCh37]Yp11.2 (7090325-7899326)∗0 (VOUS)	580	Paternal inherited	INTS4L1,INTS4L2,RSL24D1P3,ZNF92	Advanced maternal age	No apparent abnormalities
P13	M	G2P1	34W + 1	3.1	48	46, XY	arr[GRCh37]7q11.21 (64554073-65148399)∗1 (VOUS)	594	Maternal inherited	INTS4L1,INTS4L2,RSL24D1P3,ZNF92	Advanced maternal age; increased NT	No apparent abnormalities
P14	F	G1P0	39W	3.17	51	46, XX	arr[GRCh37]2q13 (110873834-110980295)∗3 (VOUS)/arr[GRCh37]7q11.21 (64543313-65148399)∗1 (VOUS)	605	Maternal inherited	INTS4L1,INTS4L2,RSL24D1P3,ZNF92	High risk NIPT result of chromosome 6	No apparent abnormalities
P15	F	G2P0	40W + 2	3.6	50	46, XX	arr[GRCh37]6q14.1 (80109532-80351666)∗3 (VOUS)/arr[GRCh37]7q11.21 (64612879-65148399)∗1 (VOUS)	536	N.A.	INTS4L1,INTS4L2,RSL24D1P3,ZNF92	Abnormal childbearing history	No apparent abnormalities
P16	M	G1P0	40W + 5	3.55	52	46, XY	arr[GRCh37]2q13 (110498141-110980295)∗3 (VOUS)/arr[GRCh37]7q11.21 (64612879-65148399)∗1 (VOUS)	536	N.A.	INTS4L1,INTS4L2,RSL24D1P3,ZNF92	Retrocaval inferior vena cava in fetal ultrasound	No apparent abnormalities
P17	N.A.	G2P1	N.A.	N.A.	N.A.	47, XN, +18	arr (18)∗3/arr[GRCh37]4q35.2 (188411408-189618403)∗1 (VOUS)/arr[GRCh37]7q11.21 (64612879-65148399)∗1 (VOUS)/arr[GRCh37]19p12q12 (21551851-30022736)∗3 (VOUS)	536	N.A.	INTS4L1,INTS4L2,RSL24D1P3,ZNF92	Advanced maternal age	Terminated pregnancy
												
												
P18		G3P1				46, XY	arr[GRCh37]5q35.3 (178730384-178922733)∗3 (VOUS)/arr[GRCh37]7q11.21 (64612879-65148399)∗1 (VOUS)	536	N.A.	INTS4L1,INTS4L2,RSL24D1P3,ZNF92	Advanced maternal age	

N.A. = not available, NIPT = noninvasive prenatal testing, NT = nuchal translucency, SNP = single nucleotide polymorphism, W = week.

The cases can be classified to 3 subgroups:

(1) pure interstitial 7q11.21 prenatal diagnosed,
(2) four parents tested after a positive antenatal test, and
(3) others with the microdeletion combined with other genetic conditions.

In subgroup 1, the 8 pure interstitial 7q11.21 microdeletion cases all had health newborns in the follow-up outcomes. The parents of fetuses P11 to P14 took SNP array analysis as well to help classify the pathogenic categories of abnormal microarray results of their fetuses. All fetuses with parental tests inherited their 7q11.21 microdeletion from their parents and all parents presented no abnormal clinical phenotype. Only the mother of P10 terminated the pregnancy due to the ultrasound abnormal fetal strephenopodia. In all combined fetuses with or without the parental test, only P11 was with likely benign CNVs and P17 was pathogenic 18 trisomy. The other combined CNVs were variant of uncertain significance. The combined microdeletions or microduplications were not the main reason for the termination of pregnancies. In subgroup 3, the mothers of P17 decided to terminate the pregnancies since P17 has a karyotype with 18 trisomy.

## 4 Discussion

In this study, we presented 18 rare prenatal cases with 7q11.21 microdeletion ranging from 517 kb to 605 kb according to SNP array. Two of them were inherited from father, while 2 were inherited from mother. To the best of our knowledge, there is no literature report about pure 7q11.21 microdeletion. Our study is the first study involving sufficient cases to discuss the pathogenicity of this deleted CNV.

Pure chromosomal 7q11.21 microdeletion is rare. In the previous study, there were patients with deletion 7q11.21-q11.23 and infantile spasms. The clinical features included atresia, bilateral radioulnar synostosis, left inguinal hernia, hypsarrhythmia, bitemporal narrowing, periorbital fullness, downslanting palpebral fissures, upturned and small nose, long philtrum, full cheeks, full lips, and severe developmental delay.^[8]^ In a study of chromosomal deletion and human malformation, the researchers found 7q11.21 associated with choanal atresia, split hand, and inguinal hernia.^[10]^ Patients with normal phenotypes can also carry this chromosomal rearrangement in the DECIPHER database.

Since the current reports about pure 7q11.21 are quite limited and inconsistent, we summarized the clinical phenotypes of patients involving or overlapping 7q11.21 microdeletions in Table 1. In 11 cases, aberrations were merely in the region of 7q11.21, ranging from 517 kb to 605 kb. Among the microdeletions, 2/18 patients were paternally inherited, 2/18 were maternally inherited, and 14/18 patients were not available. The parental tests of 4 cases showed that the partial 7q11.21 microdeletion was associated with normal phenotype. Seventeen cases presented normal karyotypes, while 1 fetus has abnormal karyotype, 47 XN +18.

Meanwhile, we summarized the comparable cases harboring pure deleted CNVs of 7q11.21 (chr7: 64543313-65196780) in the DECIPHER database (14 cases) and the ISCA database (16 cases). The proportions of pathogenicity were as follows: benign (4/30), likely benign (1/30), unknown (8/30), uncertain (7/30), uncertain: likely benign (9/30) and likely pathogenic (1/30). Despite the patients with unknown pathogenicity, the pathogenicity of these deleted regions is still uncertain. In benign cases, the patients (nssv582004, nssv706641, nssv707177, and nssv707291) presented with global developmental delay, abnormality of the nervous system, abnormal facial shape, additional significant developmental, and morphological phenotypes referred for genetic testing. In patient 289710 with likely benign CNV, the phenotypes were aggressive behavior, autism, intellectual disability, macroorchidism, and stereotypy. There were 2 variants, and the 7q11.21 microdeletion was inherited from the mother. Uncertain cases (patient 288626, 301787, 338868, 339405) had autism, intellectual disability, global developmental delay phenotype. For patients with uncertain: likely benign CNVs (nssv706717, nssv1602118, nssv1602460, nssv1604024, nssv1604249, nssv1604308, nssv1604787, nssv1604907, and nssv1604972), they had similar phenotypes with uncertain cases along with additional incoordination, hypertonia, myopathy, developmental regression, seizures. Based on the diversity of phenotypes and CNV pathogenic classifications, we can see the pathogenicity of these deleted regions need further support.

**Figure 1:**
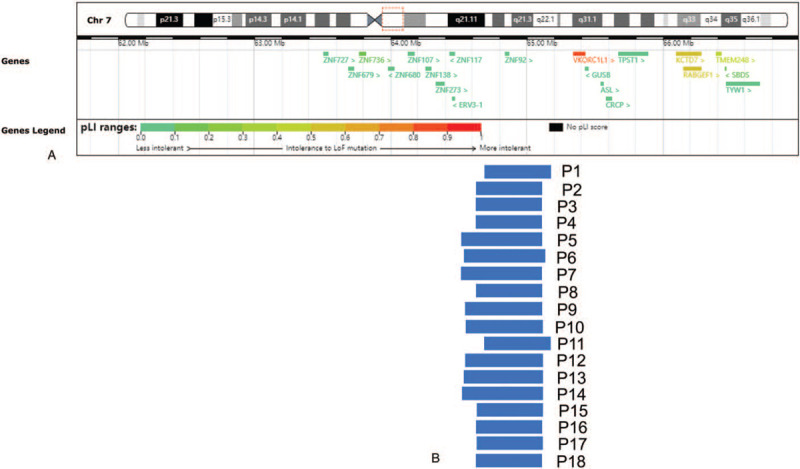
Scale representation of the duplicated region in the proximal long arm of chromosome 7 (7q11.21) (https://decipher.sanger.ac.uk/) (A) Location of morbid genes in the region. (B) Deleted fragments in the present cases in the region.

In the follow-up survey, 2 fetuses were terminated and 1 was still unborn. The rest outcomes were normal, and newborns were without apparent abnormalities. The terminations of pregnancy were either because of trisomy 18 or fetal strephenopodia in ultrasound. To further characterize the interpretations of the 7q11.21 microdeletion, we made detailed comparisons of the cases (Fig. 1).

According to the DECIPHER database, a total of 20 protein-coding genes exist in the 7q11.21 region (Table 2), which has diverse functions and is associated with various phenotypes. In this study, fetuses share similar deletions of ZNF92 (OMIM 603974; chr7:64838712-64866038) (Fig. 1). The gene *ZNF92* encodes the zinc finger protein 92, which is one of the Zinc finger proteins (ZNFs) binding nucleic acids and perform critical functions, like regulating transcription.^[16]^ ZNF92 is a member of the ZNF91 family, a subgroup of the Kruppel-associated box zinc finger proteins. The expression of this family widely appears in human tissues, especially the highest in T-lymphoid cells.^[17]^ It may be involved in transcriptional regulation.

**Table 2:** Genes in the region of 7q11.21 and the associated diseases.

Gene	OMIM	Description	Diseases
*ASL*	608310	Argininosuccinate lyase	Argininosuccinate lyase Deficiency
*CRCP*	606121	CGRP receptor component	–
*ERV3-1*	131170	Endogenous retrovirus group 3 member 1, envelope	–
*GUSB*	611499	Glucuronidase beta	–
*KCTD7*	611725	Potassium channel tetramerization domain containing 7	–
*RABGEF1*	609700	RAB guanine nucleotide exchange factor 1	–
*SBDS*	607444	SBDS ribosome maturation factor	Shwachman–Diamond syndrome
*TMEM248*	–	Transmembrane protein 248	–
*TPST1*	603125	Tyrosylprotein sulfotransferase 1	–
*TYW1*	611243	tRNA-yW synthesizing protein 1 homolog	–
*VKORC1L1*	608838	Vitamin K epoxide reductase complex subunit 1 like 1	–
*ZNF107*	603989	Zinc finger protein 107	–
*ZNF117*	194624	Zinc finger protein 117	–
*ZNF138*	604080	Zinc finger protein 138	–
*ZNF273*	604756	Zinc finger protein 273	–
*ZNF679*	–	Zinc finger protein 679	–
*ZNF680*	–	Zinc finger protein 680	–
*ZNF727*	–	Zinc finger protein 727	–
*ZNF736*	–	Zinc finger protein 736	–
*ZNF92*	603974	Zinc finger protein 92	–

OMIM = Online Mendelian Inheritance in Man.

Currently, there is no available evidence for the haploinsufficiency in association with ZNF92. The rest genes of 7q11.21 microdeletions in this study were pseudogenes, INTS4L1, INTS4L2, and RSL24D1P3. In literature, pseudogene competitively binding to shared miRs with their parental coding genes as members of the competing endogenous RNA network.^[18–20]^ Although INTS6P1 post-transcriptionally regulates the expression of INTS6 through a similar mechanism,^[21]^ the functions of pseudogenes in this study were unclear now. All fetuses in our study did not show abnormal phenotype associated with ZNF92, INTS4L1, INTS4L2, or RSL24D1P3. Moreover, P11 and P12 independently inherited the 7q11.21 from a normal father, while P13 and P14 separately inherited it from a mother without unusual clinical phenotypes. Therefore, we speculate that the partial deletion of 7q11.21 (chr7: 64543313-65196780), including ZNF92, might be a benign variant.

## 5 Conclusions

In this report, we analyzed 18 patients with similar 7q11.21 microdeletion from 517 kb to 605 kb, including ZNF92 by SNP array. The clinical utilization of CMA analysis detected additional clinically significant of submicroscopic CNVs compared with karyotyping. Our report revealed that the partial 7q11.21 deletion (chr7: 64543313-65196780) might be benign and have no association with human disorders.

## Acknowledgments

The authors would like to thank all the staff of the Genetics Laboratory, Center for Prenatal Diagnosis, First Hospital, Changchun, China for their excellent work.

## Author contributions

**Conceptualization:** Hongguo Zhang, Ruizhi Liu.

**Data curation:** Yang Yu.

**Formal analysis:** Linlin Li.

**Investigation:** Ruizhi Liu.

**Methodology:** Leilei Li.

**Project administration:** Yang Yu.

**Validation:** Yuting Jiang.

**Writing – original draft:** Hongguo Zhang.

**Writing – review & editing:** Ruizhi Liu.

## Abbreviations

Abbreviations: CMA = chromosomal microarray, CNVs = copy-number variations, OMIM = Online Mendelian Inheritance in Man, SNP = single nucleotide polymorphism.
